# On-load measurement method for the reliability of distribution transformers

**DOI:** 10.1016/j.mex.2020.101089

**Published:** 2020-10-09

**Authors:** Lambe Mutalub Adesina, Ademola Abdulkareem, Olalekan Ogunbiyi, Oladimeji Ibrahim

**Affiliations:** a Department of Electrical and Computer Engineering, Faculty of Engineering and Technology, Kwara State University, Malete, Nigeria; b Department of Electrical and Information Engineering, Covenant University, Sango Ota, Ogun State, Nigeria; c Department of Electrical and Electronics Engineering, University of Ilorin, Nigeria

**Keywords:** Cellulose deterioration, Energy analyzer, Experiment, Fluke, Insulation, Network reliability, Outages, Power quality

## Abstract

Transformer is the most important equipment used in power system. It's ensure network stability and reliability. Transformer fails over time due to several factors such as overload, poor insulation, cellulose deterioration, poor dielectric strength of the oil etc. However, transformer owners are finding it difficult to monitor the performance of distribution transformers (DT) in their place of application which results to system failure. Analyses of this distribution transformer performance required that distribution transformer be put on-load and the secondary terminals link to Power Quality and Energy Analyzer equipment (Fluke 435 Series II). This paper presents a methodology of carrying out experimental set up of on-load reliability measurements of the two distribution transformers, 500 kVA and 300 kVA, 11/0.415 kV as case study. Recommendations for the operations and maintenance engineers in charge to comply are discussed. This approach using the prescribed power quality equipment gives a reliable experimental method of evaluating the performance of distribution transformers.

• Maintaining the selected distribution transformer on – load prior to and during the experiment.

• The setting of experimental circuit to determine the operational parameters involving DT connection to Power Quality and Energy Analyzer (PQEA) equipment.

• It utilize personal computer to download the measured parameters for graphical and statistics analysis.

## Graphical abstract

**Figure:**
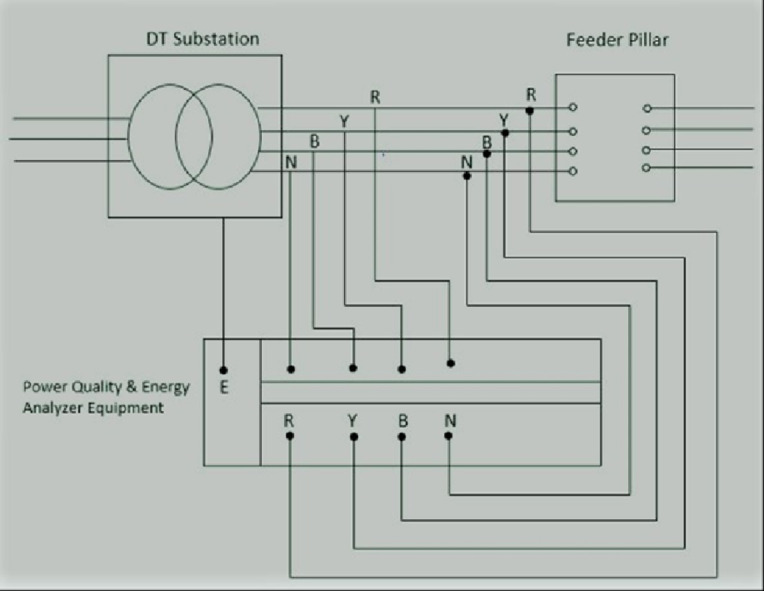

Specifications table

**Table:** 

Subject Area	Engineering
More specific subject area	Distribution Transformer Network
Method name	Distribution Transformer Reliability Measurement Experiment
Name and reference of original method	Not Applicable
Resource availability	Not Applicable

## Method details

### Background

Transformers are power system's equipment used in conversion of voltage of an alternating current (AC) supply with a corresponding conversion in current [1]. The principle of electromagnetic induction allows voltage conversion without change of supply frequency [2], [3], [4], [5], [6]. Areas of application transformers in power systems network includes; generation companies, transmission companies and distribution service providers’ networks. A transformer is made up of two compartments of different number of windings on the same magnetic core in steel laminations. These compartments are referred to as Primary side and Secondary side of transformer. Very often, there are two classes of transformer according to voltage transformation, namely; step up and step down transformer. In former, higher turns on secondary coil makes the voltage induced in the secondary coil to be larger than the lower compartment. By nomenclature, N_P_ and N_S_ denote the number of turns in primary and secondary compartments respectively and so, their Voltages are V_P_ and V_S_ [2], [3], [4], [5], [6]. In later, higher turns on the primary coil makes the voltage induced in the primary coil to be higher than the upper compartment. Thus, the same equation holds for both categories. Also, transformers at times are categorised by their core configurations as either Shell or Core. In shell, the path on the core for flux returning is externally designed to enclose the windings whereas in core the limbs of the transformer is designed be concentrically surrounded by the windings.

Generally, distribution transformers are mostly utilized by the electricity service providers because of its designed capability to transform medium voltage from primary voltage to utilization voltage. A number of methods for converting 3 phase voltage to either higher or lower voltages which are available in practise [2,5]. Very common among these connection on the field includes, star-star, delta-delta, star-delta, delta-star, open-delta and Scott connection [2], [3], [4], [5], [6]. In distribution substations, the delta-star connection transformers are commonly used by service providers in making electricity supply available to their prospective customers [2], [3], [4], [5], [6], [7], [8], [9], [10], [11], [12]. If applied as step-up transformer, the secondary compartment will be star connected; But when applied as step-down transformer, secondary compartment will be star connected because the supply of electricity to customers will require a grounded neutral (Primary distribution) [2], [3], [4], [5], [6].

In power generation analysis, the magnitude and displacement of the 3 phase output are observed to be approximately balanced compared to what is obtainable in both distribution and transmission networks [7], [8], [9], [10]. The aftermath of this is that, unbalanced Voltage for example deteriorates the operation and performance of electrical equipment such as distribution transformers [7], [8], [9], [10], [11], [12], [13], [14], [15], [16]. Consequently, this paper aims at analysing some three phase distribution transformers in a network of a power utility company having an unbalance voltage conditions. It will involve a development of an algorithm for on-load measurement method for the reliability of distribution transformers. Details of the selected distribution transformer substations used in this study would be presented while a list symbols and its descriptions are also presented.

### Distribution network operational parameters

In Nigerian practise, it has been very difficult to effectively monitor the performance of distribution transformers in the circuit of most power utility companies, perhaps because there are often too many distribution transformers on a designated feeder. Very often, percentage loading of transformer is the only parameter used to measure transformer performance in the system. This accounts for the reasons why a large number of transformers are lost in circuit. Therefore, the approach in this paper is to demonstrate the use of Power Quality and Energy Analyser equipment (Fluke 435 Series II) for an experiment that determine parameters that are useful in monitoring distribution transformers’ performance while the DT is on – load. The distribution network operational parameters which are often determine in measuring network efficiency include, power in different forms, operating frequency, phase to neutral voltage, phase to phase voltage, current flowing, power factor, harmonics etc.

Power factor relates active power to apparent power of the Network [17]. Considering distribution level, it could further be defined as a measure of active power consumed by customers to the apparent power delivered to the customers and this is referred to as electrical efficiency [18]. In alternating current circuit, phase difference exist between the supplied voltage and current flowing in the network. Thus, power factor of any power system's network is termed cosϕ, and is mathematically represented as [19];

(1) $$Cos\phi =\frac{S}{\left\{1.73\;\left(IV\right)\right\}}$$

Where,

*S* = Apparent power of the transformer in Volt-amperes.

*I* = Current flowing in the circuit.

*V*= Line voltage of the transformer.

Eq. (1) can also be re-written as [19]

(2) $$Power\;factor=\frac{kW\left(Real\;power\right)}{kVAR\;\left(Reactive\;Power\right)}$$

Since kW=(kVA)Cosϕand kVAR=(kVA)Sinϕ

Therefore,

(3) $$kVA=\;{\left[\left(KW^{2}\right)\;+\;\left(KVAR^{2}\right)\right]}^{0.5}$$

Where, kVA = Apparent power in kilovolt amperes

KW = Active power in kilowatts

KVAR = Reactive power in kilovolt amperes reactive

From Eqs. (1) to (3) above, it is evident that power factor varies with load and its value ranges from 0 to 1. A power distribution network generally comprises of both linear and non-linear loads. The apparent power (KVA) is higher in value than the active power (kW) because the nonlinear loads distorts the current wave trend drawn from the source. In power distribution network, a customer- load with high power factor draws less current when compared to customer-load with low power factor even when they are given equal power to be delivered. Thus, it is clearly evident that running network at high power factor minimizes loss in circuit than running network at low power factor. Therefore, network on low power factor that draws higher currents will definitely add to the energy lost in network. Low current rated cable will surely cost less than high current rated cable, imply that power utility company would be forced to spend huge amount in purchasing high rated cable and other equipment. Consequently, power utility companies often place a higher revenue charge to both industrial and commercial customers to compensate for the low power factor condition of the network [20].

Power factor (PF) correction leads to power factor improvement. In the course of correcting power factor, a noticeable reduction of apparent power is drawn from ac source which in turn minimizes the distribution losses. The PF corrections majorly involves the reactive power control in the distribution network [17]. Different techniques could be employed to generate reactive power to improve the network or equipment power factor [21]. Among the techniques is the reactive power compensation which is a productive technology employed in improving the performance of power systems. In modern power system, the demand for reactive power control source most time results to efficiency and reliability operation of the network. In summary, it is important to note that reactive power Compensators (VAR compensators) needs controlling to enhance reactive power supports at both static and dynamic power system operating conditions [17]. Power quality problem may arose due to voltage, current or operation frequency falling below specification levels. The economic implication of power quality for customers include the inability to use the power supplied, power utility companies (Service Provider) particularly on the accrued monthly revenue collections and lastly on power equipment malfunctioning.

### Method of experimental design for reliability measurement of the on-load distribution transformer

**Fig. 1:**
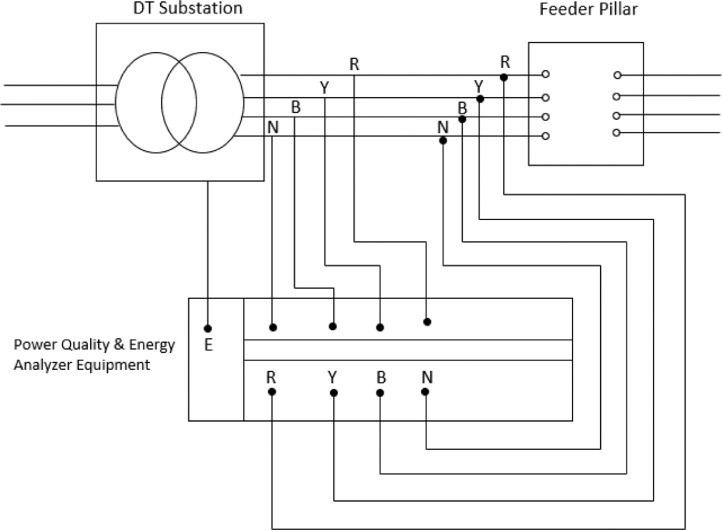
Experimental circuit arrangement for substation operational management.

The approach involves knowing or searching for either overloaded DT substations or troublesome DT substations in an electric power utility company's network or network of distribution transformers with different power ratings in circuit. The most critically overloaded DT substation is eventually selected, and its site would be visited for pre-experimental inspection. In cases where the network is very large, pre-inspection is required to know the source of supply to the substation, healthiness of both the high tension and low tension lines, Neatness of the Substation, Proper connection of substation equipment such as cable, cable lugs, bushings, earthing, feeder pillar etc., and the availability of the 33 kV or 11 kV feeder that feeds the substation of the distribution transformer selected for the experiment. This experiment is often carried out on-line i.e. while the feeder line is ON and customers on such distribution transformer are on electricity supply. The circuit diagram of the experimental connection of the Feeder line, Distribution transformer, Feeder pillar and the Testing equipment i.e. Power Quality and Energy Analyser equipment (PQEA) is shown in Fig. 1.

Two distribution transformer substations were eventually selected. This includes, a 1 × 500 kVA, 11/0.415 kV substation and a 1 × 300 kVA, 11/0.415 kV substation. At each of the DT substation, the process of connecting Fig. 1 to achieve results are carried out in the following order as shown below:

1. Ensure a fault free network and that the DT substation is on electricity power supply.
2. Connect a section of Fluke 435 to low voltage bushings of the on-line DT transformer (i.e. at the secondary terminals of the DT)
3. Connect the other section of Fluke 435 to the incomer cable terminals at the Feeder Pillar side of the substation.
4. Ensure proper Calibrate Fluke 435 Machine such that the duration time (T) of the experiment is selected and time interval (t) of recording readings is also selected. Although, these calibrated time selections may vary from individuals to individual. However, in this research work, Duration time T and interval time t were taken as 50 and 10 min for case study 1, and 60 and 10 min for case study 2 considered.
5. Grounding of both DT and the Fluke 435 (POAE) machine.
6. Carryout the settings of the operational parameters intended to be measured on Fluke 435 Machine (PQEA). For this experiment, the parameters include, root mean square (RMS value of Voltages (Single phase and 3 Phases), Current, Frequency, all forms of Power and Power Factor.
7. Use Fluke 355 (Clamp – on – ammeter) to conventionally measure the current and voltage on each of the single core low voltage cables that spanned between the DT secondary bushings and the feeder pillar. The measured values would be compared to the results of the new protocol.
8. After all the connections, Switch - ON Fluke 435 machine (PQEA).
9. Allow the Fluke 435 (PQEA) machine to remain ON for the duration time (T) earlier calibrated.
10. When time (T) setting is completed, the PQEA equipment will be disconnected and be brought to the Office from the field for further work.
11. The recorded results in PQEA equipment at interval of time (t) in duration time T for all the operational Parameters would be downloaded using a personal computer.
12. Graphical analysis of each parameter was carried out using computer excel application.

**Fig. 2:**
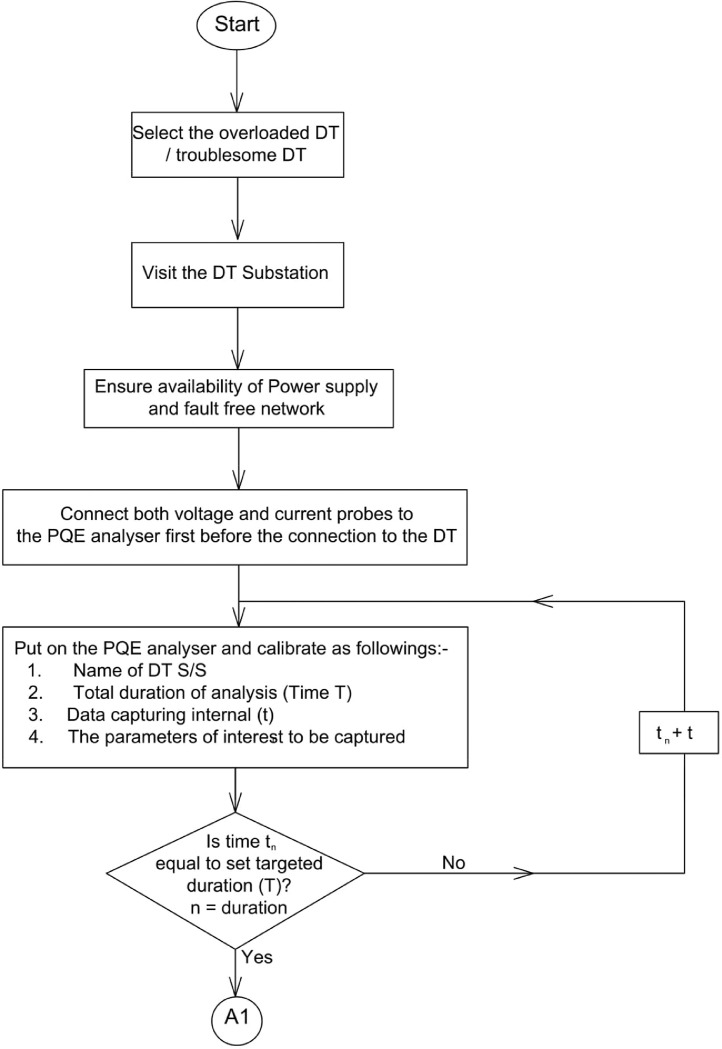

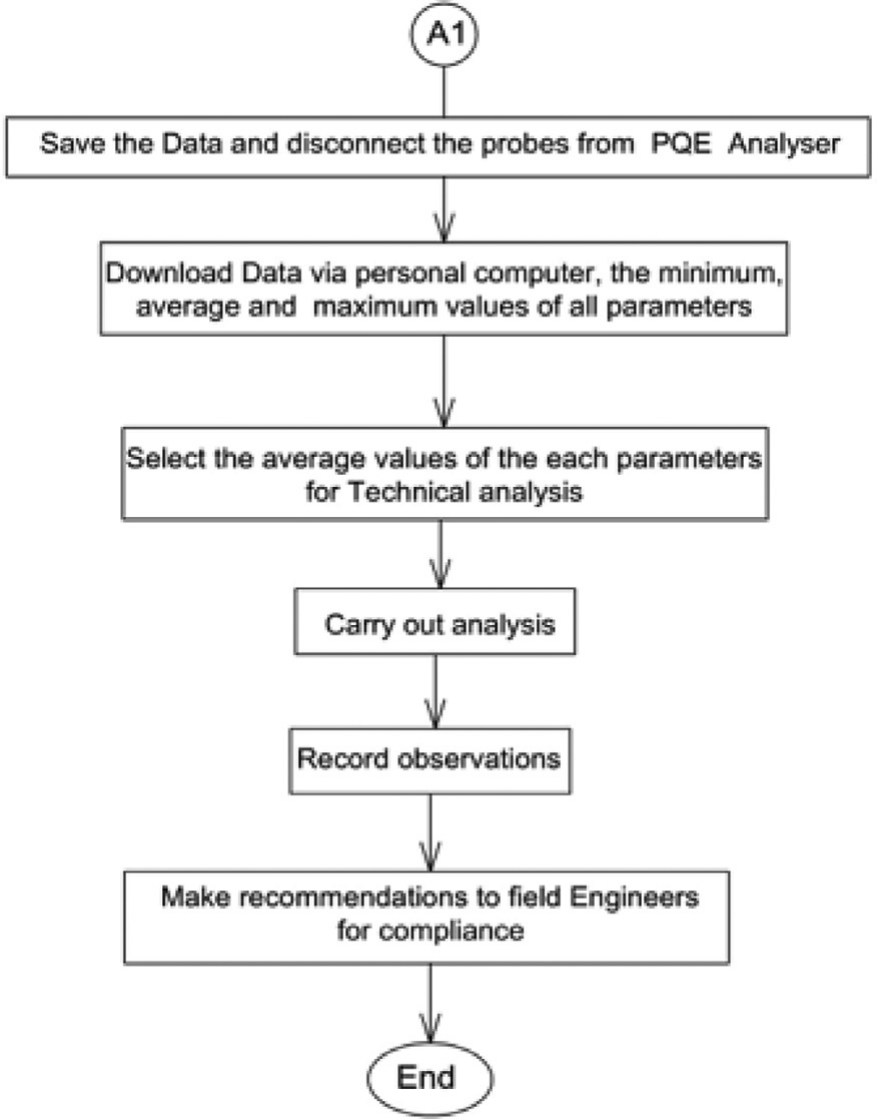
(a) Flow process of typical DT power analysis using Power Quality and Energy Analyser Equipment. (b) Continuation of Flow process of typical DT power analysis using Power Quality and Energy Analyser Equipment.

The flowchart in Fig. 2 [17,18], pictorially describe these procedural steps in detail

**Case Study 1: 500 kVA, 11/0.415** **kV Distribution Transformer Substation**

The procedures described in section above was applied to this case study, a 500kVA, 11/0.415 kV transformer substation using the under listed parameters’ calibrations.

1. Total Duration of Analysis (T1) = 50 min (for voltages, current and frequency)
2. Total Duration of Analysis (T2) = 1 hour (for AP, APR and RP and Power factor)
3. Number of readings = 6 (During voltages, currents and frequency measurements in time (T1) above)
4. Number of readings = 7 (During AP, APR and RP and Power factor measurements in time (T2) above)
5. Data Capturing time interval (t) = 10 min

The results of the experiment are presented in Table 1 to 8 while and its graphical analysis using computer excel are shown in Fig. 3 to 10.

**Table 1:** Rms voltage (phase to neutral).

Time	Vrms L1-N (V)	Vrms L2-N (V)	Vrms L3-N (V)	Vrms N-G (V)
10:11:11 AM.	219.58	232.92	233.3	1.42
10:21:11 AM.	189.64	233.42	233.54	2.06
10:31:11 AM.	159.74	233.74	234.38	2.04
10:41:11 AM.	187.22	233.12	234	1.98
10:51:11 AM.	173.08	232.92	233.6	1.96
11:11:11 AM.	167.98	231.86	232.62	2.18

**Table 2:** Rms voltage (phase to phase).

Time	Vrms L1-L2 (V)	Vrms L2-L3 (V)	Vrms L3-L1 (V)
10:11:11 AM.	392.16	401.92	393.82
10:21:11 AM.	368.54	401.92	367.72
10:31:11 AM.	343.68	403.12	343.54
10:41:11 AM.	365.22	402.44	366.36
10:51:11 AM.	353.62	401.74	353.98
11:11:11 AM.	347.86	400.26	349.14

**Table 3:** Current.

Time	L1 Current (A)	L2 Current (A)	L3 Current (A)	N Current (A)
10:11:11 AM.	154.4	164.5	231.3	77.6
10:21:11 AM.	163.9	180.4	257.4	86.5
10:31:11 AM.	165.3	179.9	225.4	59.4
10:41:11 AM.	161.5	185.1	221.8	57.1
10:51:11 AM.	171.7	172.9	238.1	67.8
11:10:11 AM.	168.3	188.8	226.5	64.9

**Table 4:** Frequency.

Time	Frequency (Hz)
10:11:11 AM.	50.389
10:21:11 AM.	50.421
10:31:11 AM.	50.446
10:41:11 AM.	50.318
10:51:11 AM.	50.377

**Table 5:** Apparent power.

Time	Apparent Power L1N (VA)	Apparent Power L2N (VA)	Apparent Power L3N (VA)	Total Apparent Power (VA)
10:11:11 AM.	33,860	38,280	53,960	126,100
10:21:11 AM.	31,060	42,080	60,100	133,240
10:31:11 AM.	26,380	42,020	52,800	121,200
10:41:11 AM.	30,160	43,140	51,880	125,180
10:51:11 AM.	29,700	40,260	55,600	125,560
11:01:11 AM.	32,040	43,080	57,440	132,560
11:11:11 AM.	28,260	43,780	52,700	124,740

Total APR = 888,580.

Ave APR = 126.94kVA.

**Table 6:** Reactive power.

Time	Reactive Power L1N (VAr)	Reactive Power L2N (VAr)	Reactive Power L3N (VAr)	Total Reactive Power (VAr)
10:11:11 AM.	6560	8440	15,940	30,940
10:21:11 AM.	4380	9140	15,720	29,240
10:31:11 AM.	3540	8840	13,740	26,120
10:41:11 AM.	4520	9340	12,560	26,420
10:51:11 AM.	4800	8120	14,380	27,300
11:01:11 AM.	4520	8560	14,940	28,020
11:11:11 AM.	4160	8540	15,060	27,760

Total RP = 195,800.

Ave RP = 27.97kVA.

**Table 7:** Active power.

Time	Active Power L1-N (W)	Active Power L2-N (W)	Active Power L3-N (W)	Total Active Power (W)
10:11:11 AM.	33,280	37,400	51,700	122,380
10:21:11 AM.	30,800	41,160	58,160	130,120
10:31:11 AM.	26,080	41,160	51,080	118,320
10:41:11 AM.	29,820	42,180	50,440	122,440
10:51:11 AM.	29,240	39,480	53,820	122,540
11:01:11 AM.	31,660	42,300	55,600	129,560
11:11:11 AM.	27,840	43,000	50,620	121,460

Total AP = 866,820.

Ave AP = 123.83 kW.

**Table 8:** Power factor.

Time	Power Factor L1N	Power Factor L2N	Power Factor L3N	Network Power Factor Total
10:11:11 AM.	0.98	0.98	0.96	0.96
10:21:11 AM.	0.99	0.98	0.97	0.96
10:31:11 AM.	0.99	0.98	0.97	0.97
10:41:11 AM.	0.99	0.98	0.97	0.97
10:51:11 AM.	0.98	0.98	0.97	0.97
11:01:11 AM.	0.99	0.98	0.97	0.97
11:11:11 AM.	0.99	0.98	0.96	0.97

Total PF = 6.77.

Ave PF = 0.97.

**Fig. 3:**
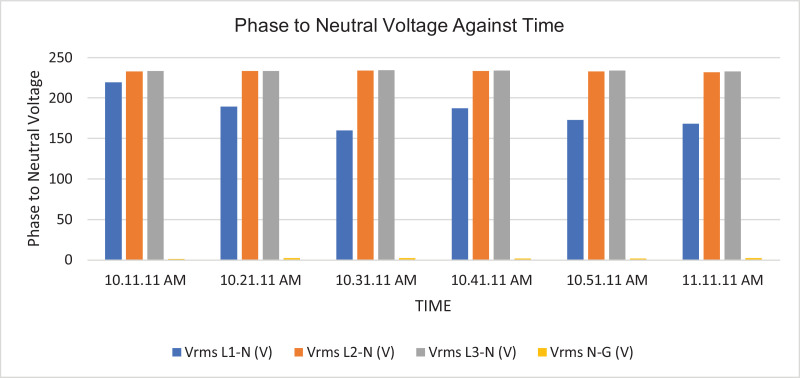
Histogram representation of Vrms (Phase to Neutral) of each phase against Time.

**Fig. 4:**
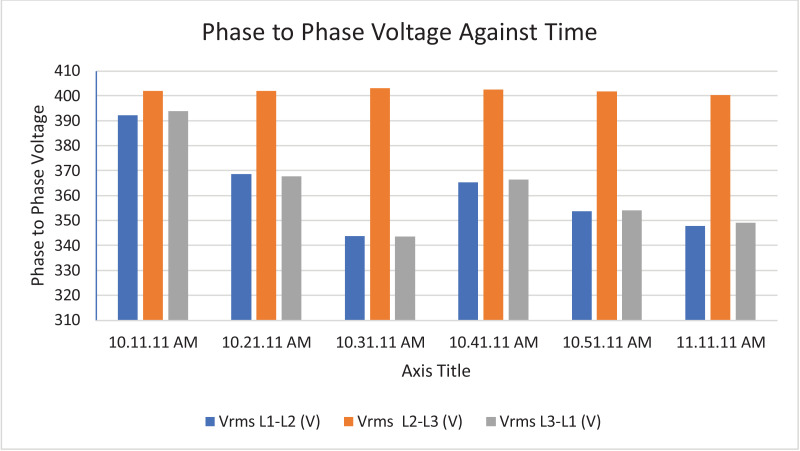
Histogram representation of Vrms (Phase to Phase) against Time.

**Fig. 5:**
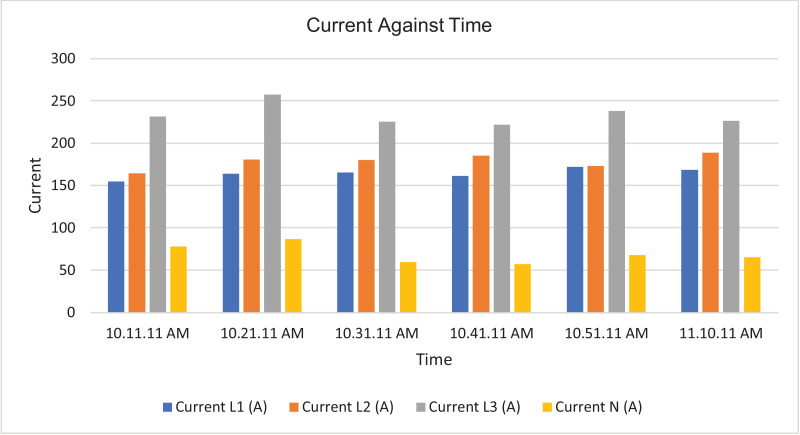
Histogram representation of current of each phase against Time.

**Fig. 6:**
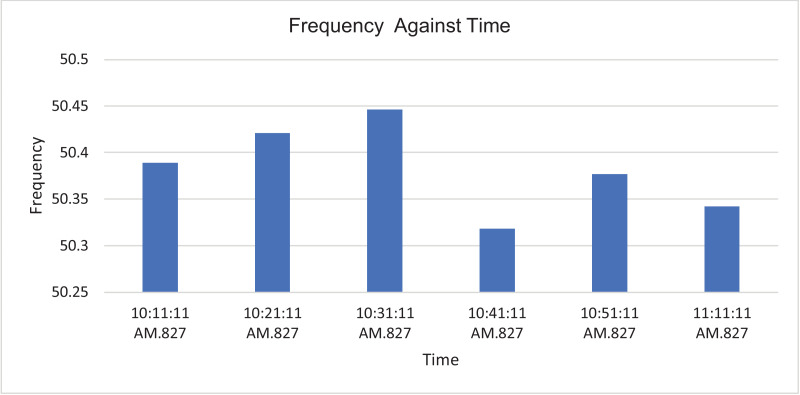
Histogram representation of Frequency of each phase against Time.

**Fig. 7:**
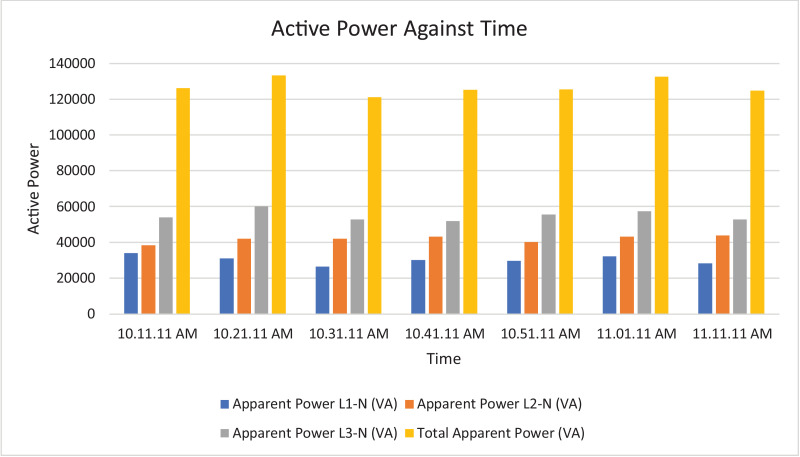
Histogram representation of apparent power of each phase against Time.

**Fig. 8:**
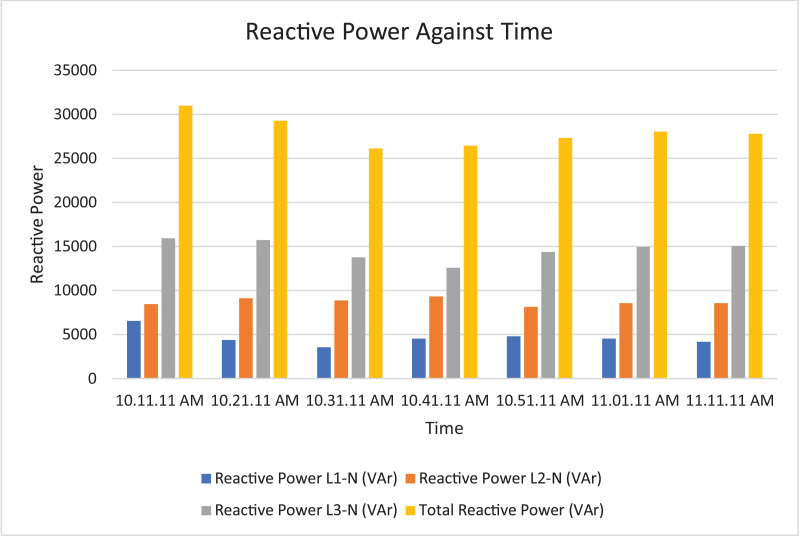
Histogram representation of Reactive power of each phase against Time.

**Fig. 9:**
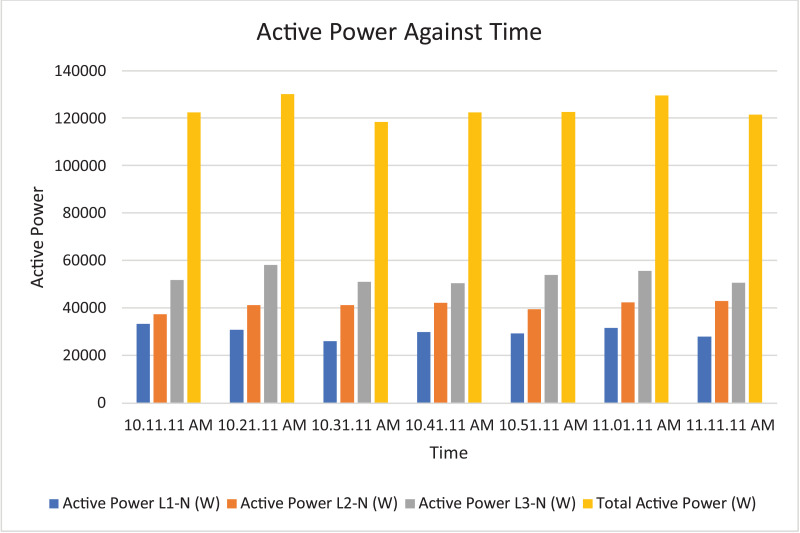
Histogram representation of Active power of each phase against Time.

**Fig. 10:**
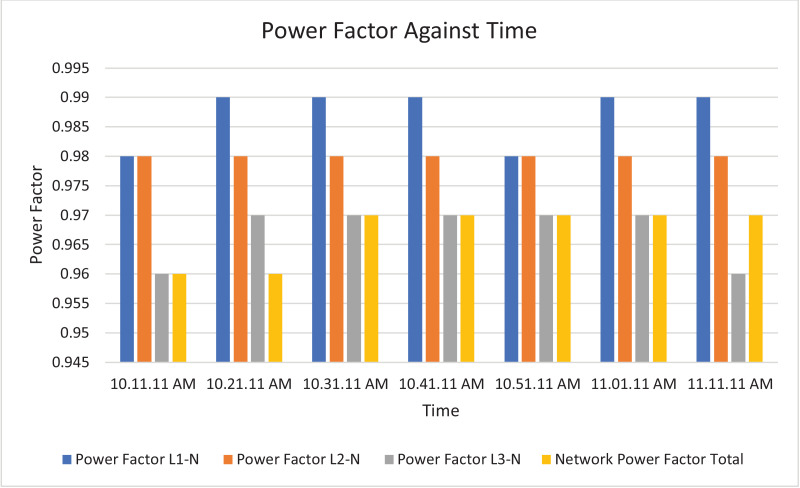
Histogram representation of Power factor of each phase against Time.

**Case Study 2: 300kVA, 11/0.415** **kV Distribution Transformer Substation**

The procedures described in section above was applied to this case study, a 300kVA, 11/0.415 kV transformer substation using the under listed parameters’ calibrations.

1. Total duration of Analysis (T) = 1 hour
2. Data capturing time interval (t) = 10 min.
3. Number of readings = 7

The results of the experiment are presented in Tables 9 to 16 while its graphical analysis using computer excel are shown in Figs. 11 to 18.

### Method validation

On case study 1, the obtained phase to neutral voltage ranges from 167.98 V to 219.58 V while the phase to phase rms voltage ranges from 343.68 V to 393.82 V. The two categories of voltage fall within the acceptable limits of standards of 230 V and 415 V respectively. The load on this transformer is not balanced because the 3 phases are not having the same values or values that are relatively closed. The operational frequency was between 50.318 Hz to 50.446 Hz, hence the operation is considered stable. The peak active power, apparent power and reactive power are 130.12 kW at 10:21:11 am, 133.24 kVA at 10:21:11 am and 30.94 kVAr at 10:11:11 am respectively. In this case, the peak power (i.e. all categories of power) did not occur the same time as it can be seen in the DT substation 2 later. The percentage of reactive power to active power in this DT network is 22.59% which is relatively alright by standards considering the values of average AP and RP. Lines Power factor is excellently alright, and they are within the range of minimum value of 0.96 and maximum value of 0.99 across the three phases. The network power factor ranges from 0.96 to 0.99 and the overall average network power factor was estimated to be 0.97. Considering the average apparent power value of 126.94 kVA loading of this DT with its 500kVA rated capacity, implies the percentage loading of this DT as at that hour was 25.39%. However, it is important to note that the readings were taken at the off-peak load period of the DT substation.

**Table 9:** Rms voltage (phase to neutral).

Time	Vrms L1-N (V)	Vrms L2-N (V)	Vrms L3-N (V)	Vrms N-G (V)
10:33:04 AM.	223.1	223.84	222.22	0.04
10:43:04 AM.	223.1	223.84	222.24	0.06
10:53:04 AM.	222.44	223.24	221.56	0.06
11:03:04 AM.	221.28	221.86	220.36	0.08
11:13:04 AM.	222.8	223.32	221.78	0.08
11:23:04 AM.	222.56	223.26	221.82	0.08
11:33:04 AM.	222.62	223.16	221.8	0.08

**Table 10:** Rms voltage (phase to phase).

Time	Vrms L1=L2 (V)	Vrms L2-L3 (V)	Vrms L3-L1 (V)
10:33:04 AM.	387.62	386.08	385.36
10:43:04 AM.	387.58	386.1	385.36
10:53:04 AM.	386.46	384.9	384.32
11:03:04 AM.	384.42	382.44	382.34
11:13:04 AM.	387.16	384.88	384.78
11:23:04 AM.	386.78	384.94	384.68
11:33:04 AM.	386.94	384.76	384.6

**Table 11:** Current.

Time	L1 Current (A)	L2 Current (A)	L3 Current (A)	N Current (A)
10:33:04 AM.	433.3	359.5	354.5	89.1
10:43:04 AM.	441.6	356.7	357.8	91.7
10:53:04 AM.	458.8	363.2	347.4	116.5
11:03:04 AM.	401.1	345.3	288.2	115.4
11:13:04 AM.	386.6	362	290.5	105.1
11:23:04 AM.	407.5	369.3	289.5	120.8
11:33:04 AM.	389.1	392.3	305.2	107.5

**Table 12:** Frequency.

Time	Frequency (Hz)
10:33:04 AM.	50.45
10:43:04 AM.	50.465
10:53:04 AM.	50.472
11:03:04 AM.	50.506
11:13:04 AM.	50.606
11:23:04 AM.	50.455
11:33:04 AM.	50.449

**Table 13:** Active power.

Time	L1-N Active Power (W)	L2-N Active Power (W)	L3-N Active Power (W)	Total Active Power (W)
10:33:04 AM.	93,480	78,120	76,400	248,000
10:43:04 AM.	95,280	77,460	77,240	249,980
10:53:04 AM.	98,740	78,700	74,560	252,000
11:03:04 AM.	85,800	74,300	61,040	221,140
11:13:04 AM.	83,240	78,620	61,980	223,840
11:23:04 AM.	87,420	79,860	61,360	228,640
11:33:04 AM.	83,280	85,220	65,000	233,500

Total AP = 1,433,900 W.

Ave. AP = 204.84 kW.

**Table 14:** Apparent power.

Time	Apparent Power L1N (VA)	Apparent Power L2N (VA)	Apparent Power L3N (VA)	Total Apparent Power (VA)
10:33:04 AM.	96,620	80,400	78,680	255,700
10:43:04 AM.	98,440	79,800	79,340	257,580
10:53:04 AM.	101,960	81,040	76,920	259,920
11:03:04 AM.	88,700	76,500	63,400	228,600
11:13:04 AM.	86,020	80,740	64,340	231,100
11:23:04 AM.	90,620	82,400	64,120	237,140
11:33:04 AM.	86,560	87,520	67,620	241,700

Total APR = 1711740VA.

Ave. APR = 244.53kVA.

**Table 15:** Reactive power.

Time	Reactive Power L1N (VAr)	Reactive Power L2N (VAr)	Reactive Power L3N (VAr)	Total Reactive Power (VAr)
10:33:04 AM.	25,600	19,780	19,700	65,080
10:43:04 AM.	25,920	19,920	19,160	65,000
10:53:04 AM.	26,680	20,100	19,840	66,620
11:03:04 AM.	23,640	18,820	17,860	60,320
11:13:04 AM.	22,740	19,160	17,980	59,880
11:23:04 AM.	24,920	21,160	19,300	65,380
11:33:04 AM.	24,600	20,740	19,380	64,720

Total RP = 447000VAr.

Ave. RP = 63.86kVAr.

**Table 16:** Power factor.

Time	Power Factor L1N	Power Factor L2N	Power Factor L3N	Network Power Factor
10:33:04 AM.	0.97	0.97	0.97	0.97
10:43:04 AM.	0.97	0.97	0.97	0.96
10:53:04 AM.	0.97	0.97	0.97	0.96
11:03:04 AM.	0.97	0.97	0.96	0.96
11:13:04 AM.	0.97	0.97	0.96	0.96
11:23:04 AM.	0.96	0.97	0.96	0.95
11:33:04 AM.	0.96	0.97	0.96	0.96

Total PF = 6.72.

Ave. PF = 0.96.

**Fig. 11:**
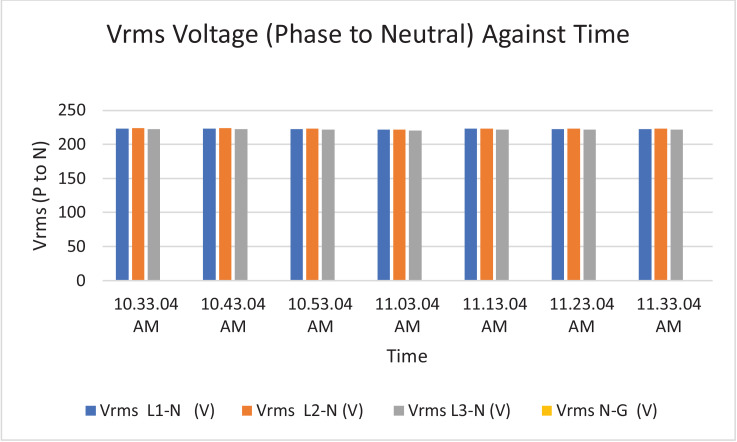
Histogram representation of Vrms (Phase to Neutral) of each phase against Time.

**Fig. 12:**
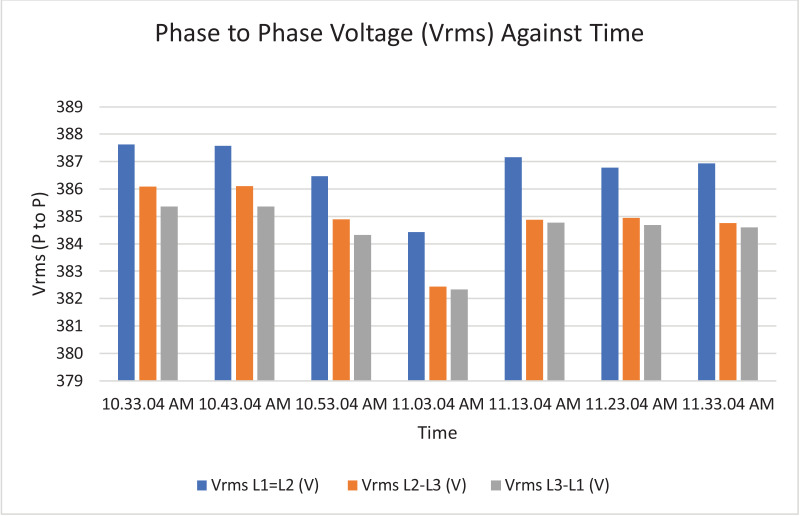
Histogram representation of Vrms (Phase to Phase) against Time.

**Fig. 13:**
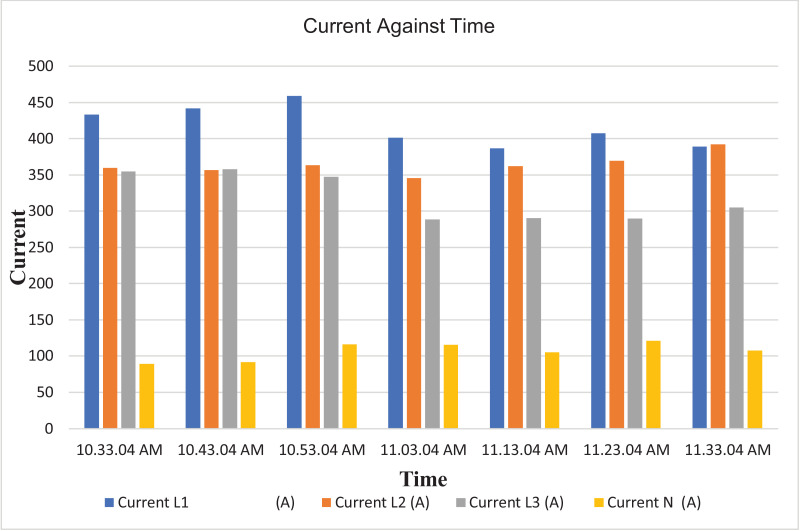
Histogram representation of current of each phase against Time.

**Fig. 14:**
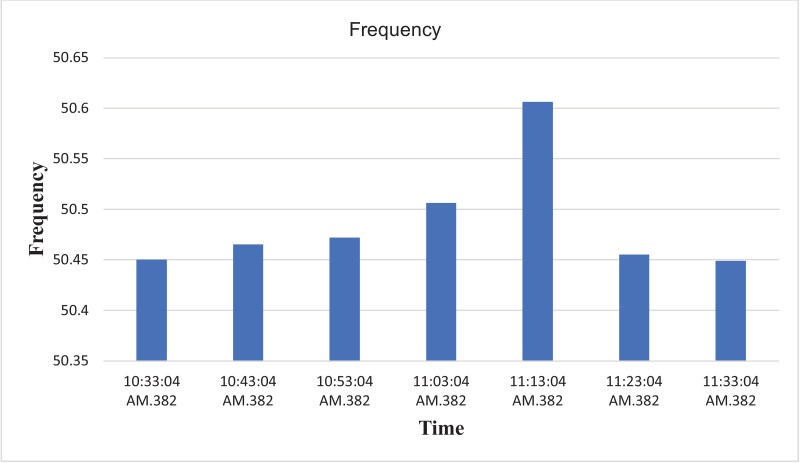
Histogram representation of Frequency of each phase against Time.

**Fig. 15:**
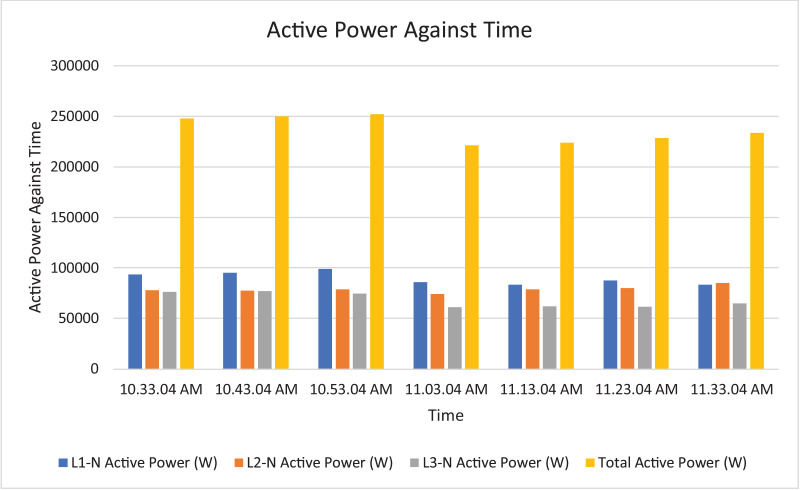
Histogram representation of Active power of each phase against Time.

**Fig. 16:**
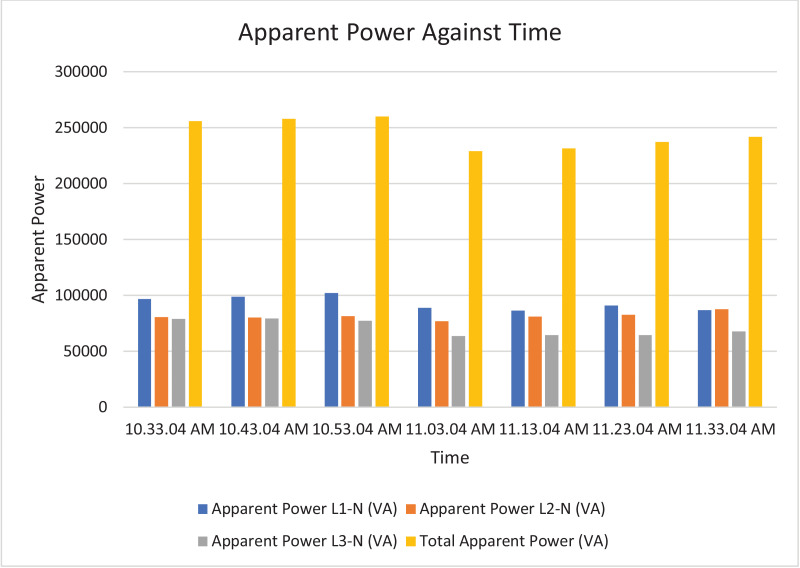
Histogram representation of apparent power of each phase against Time.

**Fig. 17:**
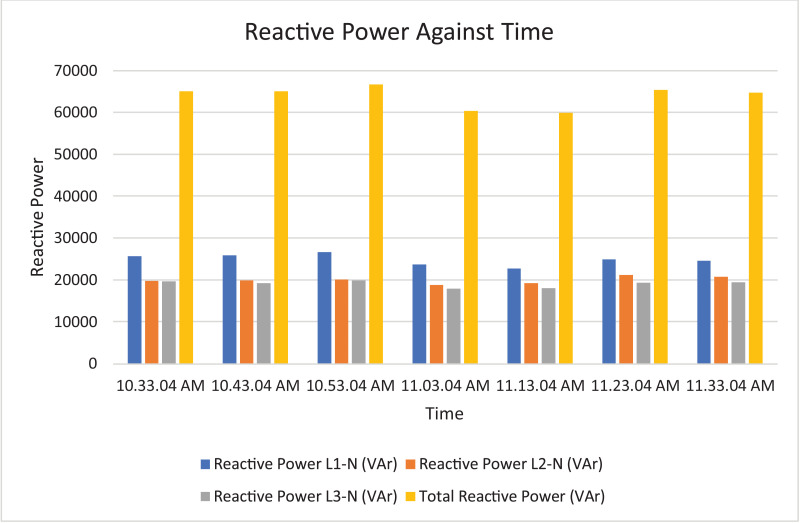
Histogram representation of Reactive power of each phase against Time.

**Fig. 18:**
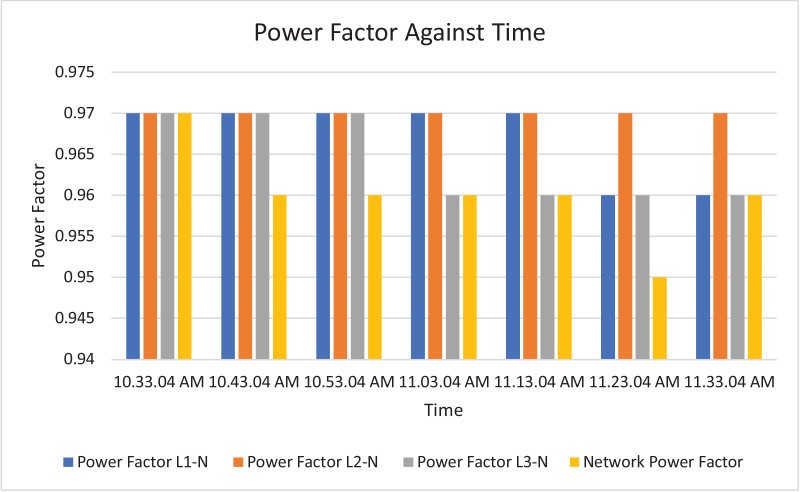
Histogram representation of Power factor of each phase against Time.

Energy index of reliability (EIR) defined as the ratio of the expected energy not served (EENS) during some long period of observation to the total energy demand (TED) during the same period. In this scenario with a system power factor of 0.97, total obtainable load on 500 kVA, 11/0.415 kV transformer is 485 kW. Transformer allowable loads is 80% which translate to 388 kWhr. TED is 130.12 kW while EENS becomes 485 kW – 130.12 kW = 354.88 kW. Therefore, the energy index of reliability is 2.73. This implies that the load on the transformer is relatively too small compared to the size of the transformer. This means the transformer is grossly under-utilized.

On case study 2, the phase to neutral voltage recorded ranges from 221.28 V to 223.84 V which falls below the conventional value of 230 V according to Nigerian Standard and Specification. The phase to phase rms voltage recorded ranges from 382.34 V to 387.62 V which falls below the convectional value of 400 V according to standard. The load on this transformer can be said to be unbalanced because the 3 phases are hot having the same values or values that are relatively closed. The frequency is measured to have ranged between 50.449 Hz to 50.606 Hz, hence the operation is also considered stable. However, ± 2% of 50 Hz frequency is allowed as standard for power system operation. Peak active power, apparent power and reactive power are 252.0 kW at 10:53:04 am, 259.92 kVA at 10:53:04 am and 66.62 kVAr at 10:53:04 am respectively. While the average power drawn from the DT at that hour of the day are 204.84 kW, 244.53 kVA, 63.86 kVAr for active power, apparent power and reactive power respectively. It imply that the percentage reactive power to active power in this network is 31.18% which violated the allowable value of 5% by IEEE regulation. Lines Power factor is satisfactorily within minimum value of 0.96 and maximum value of 0.97 across the three phases. While the network power factor ranges from 0.95 to 0.97 and the overall average network power factor was also estimated to be 0.97. Considering the average apparent power value of 259.92 kVA loading of the Distribution transformer with its 300kVA rated capacity, it implies the percentage loading of the DT as that time was 86.64%. However, it's important to note that the readings were taken at the off-peak load period of the DT substation, but the loads on it has a greater value of reactive power. It's expected that the reactive power presence would be higher at peak load period with appreciable consequence of power factor value reduction. Also, in this scenario with a system power factor of 0.97, total obtainable load on 300 kVA, 11/0.415 kV transformer is 291 kW. Transformer allowable loads is 80% which translate to 232.8 kWhr. TED is 204.84 kW while EENS becomes 291 kW – 204.84 kW = 86.16 kW. Therefore, the energy index of reliability is 0.42. This also implies the transformer is relatively loaded and can still accommodate loads of 42% rated capacity of the transformer.

### Post experimental recommendations

After the completion of the experiment and the results are obtained. It becomes necessary to pass recommendations to the owner of the distribution transformer and network at large on how the network reliability can be further improved. Such feedbacks are acted upon by the field operational engineers. Thus, these recommendations include,

1. Due to unbalanced loads in both cases of the Distribution transformer substation studied which necessitated high records of Neutral current as shown in Tables 3 and 11; it becomes necessary for the operation Engineers to comb the network and approximately balance load in all the phases.

Theoretically, the 3 phase's loads should be equal which results in zero Neutral current. In practise, this is difficult to achieve. Hence there exist minimum Neutral current that guarantee an approximate balance of 3phases [7], [8], [9].

2. The average power factor for the two distribution transformers are 0.97 and 0.96 as shown in Tables 8 and 16 respectively. Very often, when the system or network power factor falls below 0.85 on the field [17,18], installation of appropriate size of capacitor bank is required for the correction of power factor to desire specification (0.9 to 0.95).
3. Use of standard equipment and materials in the substation.
4. Proper grounding of substation equipment.
5. The distribution transformer substation must be cleaned and free from water-log.

## Conclusion

Transformer operational performance measurement should be considered a routine assignment for power utility company, manufacturing industries, corporate organisations and other private owners of transformer. This paper has presented an On-load measurement methods for the reliability of distribution transformers. It involves a development of algorithm and formulation of flowchart that described the experimental procedures. The two case studied public distribution transformers belonging to a Nigerian power utility companies generated different results which are presented and extensively discussed. Power factor determination for the transformers are most essential parts of these results. Where the determined power factor is high, a need for power factor correction installation would be required. Furthermore, some post experimental recommendations are presented to the advantage of transformer owners. From this experimental method, it is clear that on-line monitoring of distribution transformers’ performance would enhance high reliability performance of such transformers.

List of Symbols and Representations.

**Table:** 

S/N	SYMBOL	REPRESENTATION
1	L1	Phase 1
2	L2	Phase 2
3	L3	Phase 3
4	N_P_	Number of turns on Transformer Primary compartment
5	N_S_	Number of turns on Transformer Secondary compartment
6	T	Duration of Analysis
7	Vrms	Root Mean Square Voltage
8	Ph	Phase
9	N	Neutral
10	PQEA	Power Quality and Energy Analyser
11	AC	Alternating Current
12	DT	Distribution Transformer
13	RMS	Root Mean Square
14	kW	Kilowatt
15	NG	Neutral Grounding
16	V	Voltage
17	APR	Apparent Power
18	Var	Voltampere reactive
19	PF	Power Factor
20	VA	Voltampere
21	AP	Active Power
22	W	Watt
23	S/S	Substation
24	A	Ampere
25	RP	Reactive Power
26	Ave	Average

## Declarations

 

## Funding

Not applicable

## Availability of data and materials

Not applicable

## Authors’ contributions

The research work was jointly carried out by listed authors. The experimental design and sourcing for the network of 11 kV distribution transformers that were used was handled by LM. However, LM, AA, OO and OI work in collaboration to carry out the experiment at the chosen venue. OO also supervised the collation of the results. Post experimental report was drafted by AA while LM developed the manuscript for publication in an academic journal. Meanwhile, all authors read and gave consent approval to this manuscript submission.

## Declaration of Competing Interest

The authors declare that we have no competing interests.
